# Evaluation of BCG Vaccination and Plasma Amyloid: A Prospective, Pilot Study with Implications for Alzheimer’s Disease

**DOI:** 10.3390/microorganisms10020424

**Published:** 2022-02-12

**Authors:** Coad Thomas Dow, Charles L. Greenblatt, Edward D. Chan, Jordan F. Dow

**Affiliations:** 1 Department of Ophthalmology and Visual Sciences, McPherson Eye Research Institute, University of Wisconsin-Madison, Madison, WI 53706, USA; 2 Mindful Diagnostics and Therapeutics, Eau Claire, WI 54701, USA; 3 Department of Microbiology and Molecular Genetics, Hebrew University, Jerusalem 9103401, Israel; charlesg@ekmd.huji.ac.il; 4 Department of Academic Affairs, National Jewish Health, Denver, CO 80218, USA; ChanE@NJHealth.org; 5 Division of Pulmonary Sciences and Critical Care Medicine, University of Colorado Anschutz Medical Campus, Aurora, CO 80217, USA; 6 Rocky Mountain Regional Veterans Affairs Medical Center, Aurora, CO 80045, USA; 7 Mayo Clinic College of Medicine and Science, Rochester, MN 55905, USA; Dow.Jordan@mayo.edu; 8 Northwestern Wisconsin Region Mayo Clinic Health System, Eau Claire, WI 54703, USA

**Keywords:** Bacillus Calmette–Guérin, BCG, plasma amyloid, Alzheimer’s, amyloid probability score (APS), amyloid 42/40, non-specific effects of vaccines, CMV, immune risk profile, CD4:CD8, Alzheimer’s biomarker

## Abstract

BCG vaccine has been used for 100 years to prevent tuberculosis. Not all countries, including the United States, adopted the initial World Health Organization recommendation to use BCG. Moreover, many Western countries that had routinely used BCG have discontinued its use. Recent population studies demonstrate lower prevalence of Alzheimer’s disease (AD) in countries with high BCG coverage. Intravesicular instillation of BCG is also used to treat bladder cancer that has not invaded the bladder muscle wall and has been shown to reduce recurrence. Several retrospective studies of bladder cancer patients demonstrated that BCG treatment was associated with a significantly reduced risk of developing AD. Plasma amyloid β assessment has become a fertile area of study for an AD biomarker that is predictive of a positive amyloid PET scan. Mass spectrometry-based plasma amyloid 42/40 ratio has proven to be accurate and robust, and when combined with age and ApoE, is shown to accurately predict current and future brain amyloid status. These parameters, amyloid 42/40 ratio, age and ApoE genotype are incorporated into an Amyloid Probability Score (APS)–a score that identifies low, intermediate or high risk of having a PET scan positive for cerebral amyloid. Community recruitment was used for this open-label pilot study. Forty-nine BCG-naïve, immunocompetent individuals completed our study: prior to BCG prime and boost, as determined by the APS, 34 had low risk (APS 0–35), 5 had intermediate risk (APS 36–57) and 10 had high risk (APS 58–100). The APS range for the participant group was 0 to 94. Follow-up plasma amyloid testing 9 months after vaccination revealed a reduction in the APS in all the risk groups: low risk group (*p* = 0. 37), intermediate risk group (*p* = 0.13) and the high-risk group (statistically significant, *p* = 0.016). Greater benefit was seen in younger participants and those with the highest risk. The small number of participants and the nascent status of plasma amyloid testing will rightfully temper embracement of these results. However, both the favorable direction of change after BCG as well as the utility of the APS—a valuable surrogate AD biomarker—may prompt a definitive large-scale multicenter investigation of BCG and AD risk as determined by plasma amyloid peptide ratios and APS.

## 1. Introduction

The Bacillus Calmette–Guérin (BCG) vaccine has not only been viewed as an important intervention in tuberculosis control but as an adjuvant for other antigens and as a “pro-inflammatory” agent in cancer therapy. Very shortly after its introduction in humans, Calmette noted its effect, not just on the incidence of tuberculosis, but also on the general mortality of infants [1]. During the writing of this manuscript, in Lille France a conference was held to celebrate the 100th anniversary of BCG [2]. At this meeting, papers presented can be seen as a reminder of the amazing breadth of action of this attenuated bacterium in human disease and conditions. Childhood application was emphasized and a wide range of immunization methods were given. BCG not only appeared as its original self, but as a recombinant organism ready to do more and do it better. Special note was on allergies, and inflammatory and autoimmune diseases. Of special importance to our research, a paper at the conference cited its benefit in the reduction of the risk to developing Alzheimer’s Disease (AD) in BCG recipients with superficial bladder cancer [3]. The work in cancer patients was suggested by the lower levels of dementia in countries with universal BCG coverage [4]. In animal models, it was of great interest to us that BCG “reversed the cognitive decline” [5]. However, it should be noted that the mouse brain amyloid was not affected. In the human studies, instillation of BCG into the bladder would require a rethinking of this approach for the prevention of AD as a public health measure. The work presented here is an attempt to determine if two intradermal injections of BCG would affect the blood levels of Aβ42 and Aβ40.

Increasingly understood is that the pathology of AD predates clinical symptoms by decades [6,7]. Accurate blood-based tests for brain Aβ pathology are urgently needed to identify at-risk individuals and give the opportunity for disease modifying intervention in the preclinical stages of AD. A method has emerged that assesses plasma levels of Aβ42 and Aβ40. This method, from the Bateman laboratory at Washington University, immunoprecipitates Aβ to isolate it from plasma; this is followed by liquid chromatography–mass spectrometry (LC-MS) to determine Aβ42 and Aβ40 concentrations [8]. Aβ42 is lower in CSF and blood as this “stickier” form of the protein builds up in the plaques. Aβ40, being more hydrophilic, is used as the denominator in a ratio with Aβ42 to normalize inter-individual differences in Aβ production [9]. Thus, a lower Aβ42/Aβ40 ratio is considered to correlate with a greater risk of cerebral Aβ deposition.

With the LC-MS method, receiver operating characteristic analysis show that prediction of amyloid PET status by plasma Aβ42/Aβ40 ratio resulted in an area under the curve (AUC) of 0.88. When age and APOE e4 status was added to the model, the AUC went up to 0.94. Moreover, in a small subset of individuals who were negative for amyloid on PET imaging at baseline, the plasma assay predicted future amyloid positivity on PET imaging [8]. Plasma Aβ42/Aβ40 ratio with age and APOE status have been combined into an Amyloid Probability Score (APS) [10].

## 2. Methods

### 2.1. Recruitment

Community recruitment for individuals with a family history of “dementia” and 50–80 years of age drew 49 BCG naïve, immunocompetent individuals who participated and completed the study (Clinicaltrials.gov identifier: NCT04449926). Participant demographics are found in Table 1. Neurocognitive testing with the SAGE test (Self-Administered Gerocognitive Exam) revealed normal cognition (SAGE score > 17/22) in all participants except one. After giving informed consent, participants had peripheral blood drawn for basic lymphocyte profile, cytomegalovirus (CMV) IgG, and interleukin-6 (IL-6). Additional peripheral blood samples were centrifuged at room temperature or 4 °C within 30–60 min of phlebotomy. Plasma was aliquoted (0.5–1.0 mL) into polypropylene tubes and frozen at −70 to −80 °C within 2 h of phlebotomy. All samples were de-identified, shipped on dry ice to C_2_N Diagnostics, and analyzed in a blinded manner. The samples were tested utilizing liquid chromatography-mass spectrometry as previously described [8]. Additionally, an Amyloid Probability Score (APS) was determined for each participant by C2N Diagnostics based upon plasma Aβ42/Aβ40 ratio, age and ApoE status [10]. All participants had prime and boost vaccination with BCG Tice strain. FDA waiver was obtained for this use of BCG. The vaccine was reconstituted according to the package insert. A vial containing 1 × 10^8^ CFU of lyophilized BCG was reconstituted in 50 mL of saline. A single dose consisting of 0.1 mL (2 × 10^5^ CFU) was administered by slow intradermal injection using a 25 gauge/0.5 mm syringe in the deltoid area. A follow up booster dose was given 1 month after the initial dose. All participants had an anticipated local inflammatory reaction at the vaccination site. None experienced axillary lymphadenopathy nor fever. Participants returned 9 months after the initial vaccination for follow up testing of lymphocyte profile, Il-6 and plasma Aβ42 and Aβ40.

### 2.2. Statistical Analysis

Numerical data were expressed as mean ± standard deviation (SD) and median with interquartile range (IQR). Categorical data were expressed as frequency and percentages. The significance of difference of numerical data between two independent groups was assessed using parametric independent *t*-test or non-parametric Mann–Whitney U test. Welch’s correction was used for two-sample independent *t*-test when groups had unequal variances. The significance of difference of numerical data between two dependent groups was assessed using parametric paired *t*-test or non-parametric Wilcoxon–Pratt Signed-Rank Test. On the other hand, Kruskal–Wallis test was used for assessing the significance of difference of numerical data among more than two groups. The significance of difference in categorical data was assessed using Chi-square test. Statistical analysis was performed using R v. 4.1.2 (2021): A Language and Environment for Statistical Computing (R Foundation for Statistical Computing, Vienna, Austria) using RStudio 2021.09.1 as an Integrated Development Environment, (Boston, MA, USA) and GraphPad Prism v. 9.3 (GraphPad Software, San Diego, CA, USA). *p*-values < 0.05 were considered significant.

## 3. Results

### 3.1. Demographics, CMV Antibody Titer, and ApoE4 Allele

The study cohort consisted of 49 participants, of which 28 (57.1%) were female and 21 (42.9%) were males (Table 1). The average age of males was 65.7 years and was not significantly different (*p* = 0.52) from that of female participants (64.3 years). Similarly, the length of education of participants was not statistically significant between males and females (*p* = 0.47). The proportion of patients who tested positive for CMV, defined as IgG titer greater than 0.6 U/mL, was not significantly different between females and males (32.1% vs. 28.6%, *p* = 1). Although the proportion of male participants who had at least one ApoE4 allele was higher than that of female participants, the difference was not statistically significant (57.1% vs. 35.7%, *p* = 0.23). The SAGE scores were not significantly different between females and males either before or after BCG vaccine administration.

### 3.2. BCG Significantly Increased the Aβ42/40 Ratio

**Figure 1:**
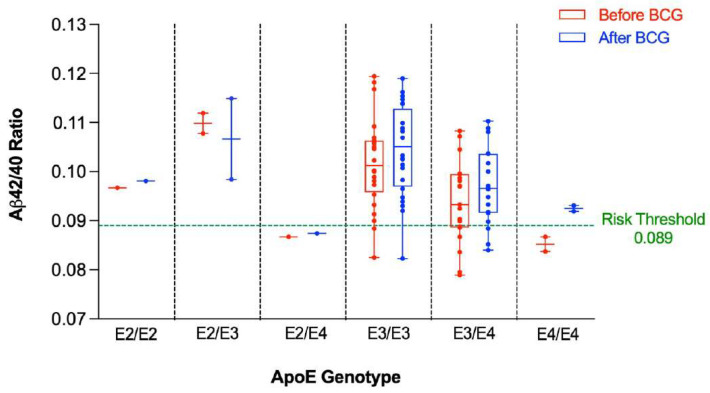
Aβ42/40 ratio before and after BCG vaccination stratified by ApoE genotype. An Aβ42/40 ratio below 0.089 is associated with higher risk of positive amyloid PET scan.

**Figure 2:**
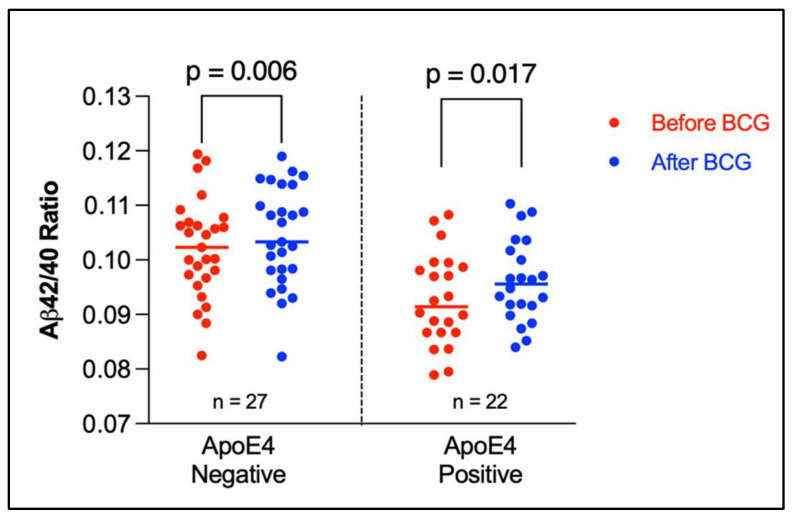
Amyloid 42/40 ratio before and after BCG vaccine administration stratified by ApoE4 alleles. The plot shows the individual values with median (horizontal segment). The number (*n*) denotes the number of observations. Statistical analysis was completed using Wilcoxon–Pratt Signed-Rank Test.

The Aβ42/40 ratio for each participant was stratified by ApoE genotype (Figure 1). As can be seen, participants having at least one ApoE4 allele tended to have lower Aβ42/40 ratio compared to participants having other alleles. Figure 2 depicts a dot plot for individual values of amyloid 42/40 ratio in participants stratified by ApoE genotype. The figure shows that participants tended to have higher values of Aβ42/Aβ40 ratio after BCG vaccine administration compared to the baseline ratio before BCG vaccine administration regardless of ApoE4 allele status.

### 3.3. Younger Participants Had More of a “Favorable” Increase in Their Aβ42/40 Ratio Than Older Participants

**Figure 3:**
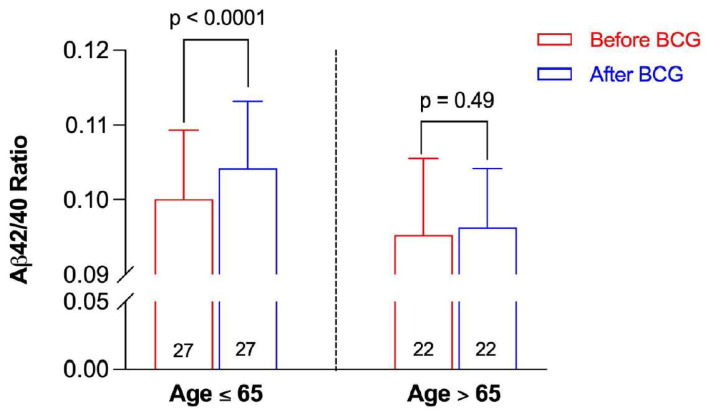
Aβ42/40 ratio before and after BCG vaccination stratified by age group. Data are presented as mean ± SD. The numbers inside the bars represent the number of observations. Statistical analysis was completed using a paired *t*-test.

When the Aβ42/40 ratio before and after BCG administration was stratified by age, participants ≤ 65 years old had a significant increase in the Aβ42/40 ratio post BCG vaccine compared to the baseline ratio before vaccine administration (0.10417 ± 0.0092 vs. 0.10006 ± 0.00901, *p* < 0.0001) (Figure 3). On the other hand, there was a non-significant increase in the Aβ42/40 ratio after BCG vaccination in older individuals > 65 years old (0.09628 ± 0.00785 vs. 0.09528 ± 0.01021, *p* = 0.49).

### 3.4. High Risk Participants Had More of a “Favorable” Decrease in Their APS Than Intermediate Risk and Low Risk Participants

**Figure 4:**
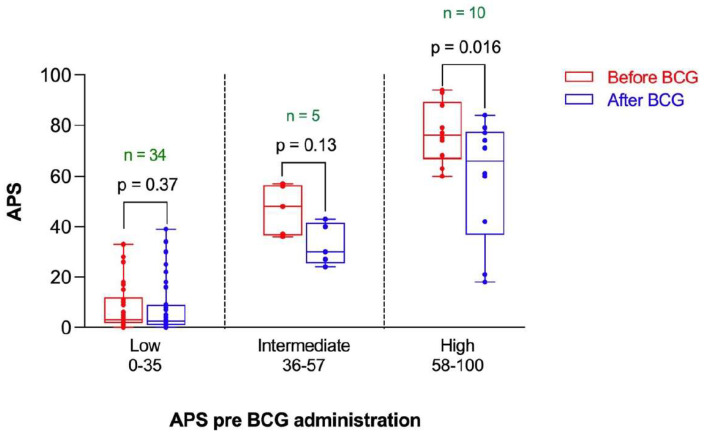
Amyloid Probability Score (APS) before and after BCG vaccination stratified by risk category based on APS pre-BCG administration. ***n*** represents the number of observations in each category. Statistical analysis was completed using a Wilcoxon–Pratt Signed-Rank Test.

The change in APS following BCG vaccination in the participants was stratified by the risk of positive PET scan as assessed by the baseline APS. The APS was reduced after BCG administration in the three risk categories (Figure 4). However, such a reduction reached statistical significance only in the high-risk category (*p* = 0.016) (Figure 4).

### 3.5. Participants without Latent CMV Infection Had a “Favorable” Decrease in Their APS with BCG Compared to Participants Latently Infected with CMV

CMV is part of the herpes family of viruses, its designation HHV-5. CMV is mostly known as an opportunist causing life-threatening infection in HIV infected persons or in immunosuppressed organ transplant recipients. In immune competent individuals, infection with CMV is usually asymptomatic; once established, its containment becomes a priority for the immune system, which is unable completely to eliminate it and its continued presence promotes age-like immune changes [11,12]. The persistent presence of CMV diverts an extraordinary amount of the T-cell resource to keep this virus in check and in doing so results in a high immune risk profile (IRP) inverting the normal CD4/CD8 ratio (normally about 2:1). CMV has been implicated as a causal agent in AD [13,14]. While another study suggests that CMV carriage facilitates HSV1-associated AD [15].

**Figure 5:**
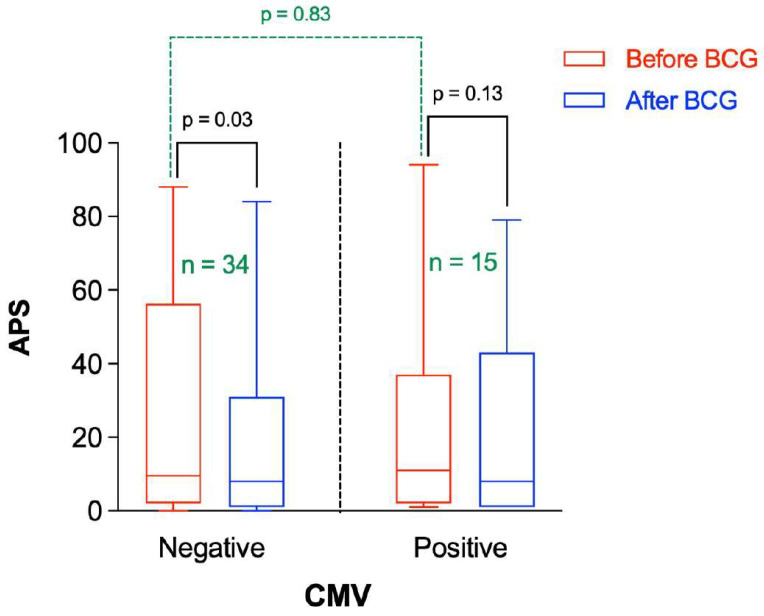
Amyloid Probability Score (APS) before and after BCG vaccination stratified by CMV status. “*n*” represents the number of observations in each category. Statistical analysis was completed using a Wilcoxon–Pratt Signed-Rank Test for dependent comparisons (solid black) or Mann–Whitney Test for independent comparisons (dashed green).

Thus, the change in APS following BCG vaccination was stratified by the CMV IgG status. There was no significant difference in APS before BCG administration between participants having negative or positive CMV IgG (Figure 5). However, the APS was significantly reduced after BCG vaccination in participants having negative CMV IgG (*p* = 0.03), whereas there was no significant change in the APS following BCG vaccination in participants with positive CMV IgG (*p* = 0.13).

### 3.6. Participants with a Higher CD4/CD8 Ratio Had a Greater Decrease in APS following BCG Vaccination Compared to Those with Lower Baseline CD4/CD8 Ratio

**Figure 6:**
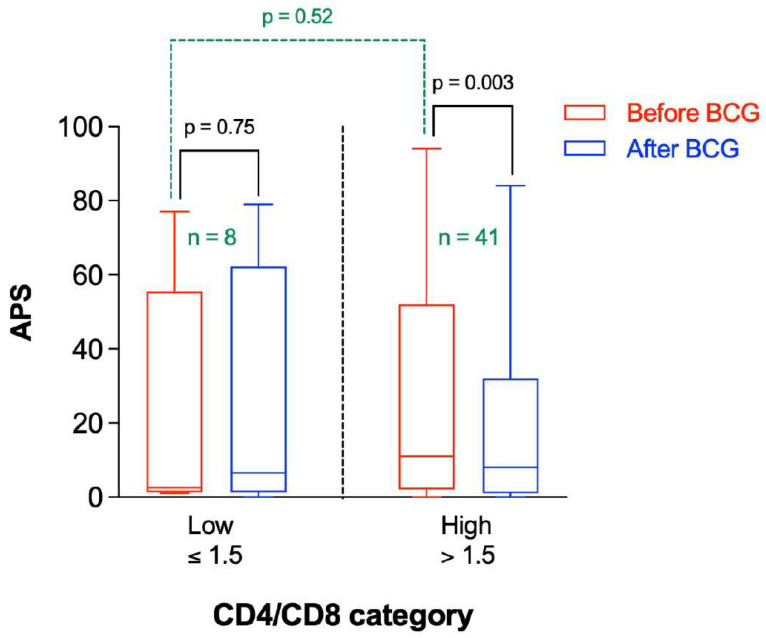
Amyloid Probability Score (APS) before and after BCG vaccination stratified by CD4/CD8 category. *n* represents the number of observations in each category. Statistical analysis was completed using a Wilcoxon–Pratt Signed-Rank Test for dependent comparisons (solid black) or Mann–Whitney Test for independent comparisons (dashed green).

There is a marked decrease in naïve CD4 subsets in patients with AD consistent with the immune system challenged by immunosenescence and/or persistent antigenic challenge [16]. The APS before and after BCG vaccination were stratified by the relative CD4/CD8 ratio (Figure 6). As can be seen, there was no significant difference in APS values before BCG vaccine administration between participants having low or high CD4/CD8 ratio (*p* = 0.52). On average, the APS was significantly lower after BCG administration in participants having a higher CD4/CD8 ratio (*p* = 0.003), whereas it was not significantly different in participants with a lower CD4/CD8 ratio (*p* = 0.75) (Figure 6).

### 3.7. Participants without Latent CMV Infection Had a More “Favorable” “Immune Risk Profile” Than Those with Latent CMV

**Figure 7:**
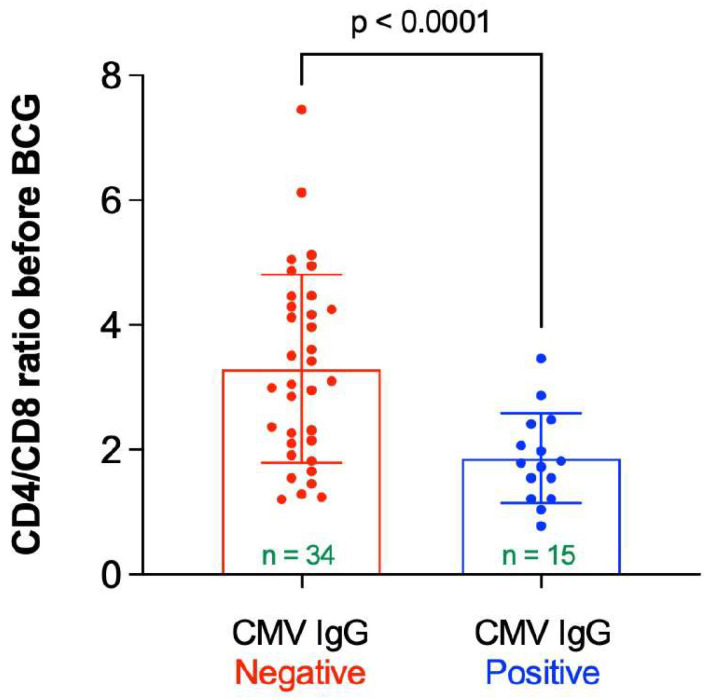
CD4/CD8 ratio before BCG vaccination stratified by status of CMV IgG. “*n*” represents the number of observations in each category. Statistical analysis was completed using Welch’s *t*-test.

In elderly individuals positive for CMV there is paucity of naïve T-cells and an accumulation of terminal T-cells with no proliferative capacity [17]. This “immune risk profile” manifests in an inverted CD4:CD8 ratio [17,18]. Thus, the mean CD4/CD8 ratio was measured as a function of the CMV IgG status. As can be seen in Figure 7, the mean CD4/CD8 ratio before vaccine administration was significantly higher in participants having negative CMV IgG compared to participants having positive CMV IgG (mean ± SD = 3.30 ± 1.51 vs. 1.87 ± 0.19, *p* < 0.0001). The mean CD4/CD8 ratio was not significantly altered in participants following BCG vaccine administration compared to the baseline value (2.86 ± 1.47 vs. 2.84 ± 1.47, *p* = 0.716).

## 4. Discussion

In this study, the reduction of risk of cerebral amyloid as determined by increase in plasma Aβ42/40 ratio and decrease in APS after BCG vaccination is reported. The favorable changes are statistically significant in the younger participants, in those with the highest risk category, those not latently infected with CMV and those participants with a favorable lymphocyte immune risk profile (CD4:CD8 > 1.5).

Integral to the study, determination of the amyloid beta peptides by the methods of Schindler et al. of the Bateman laboratory is based on isolation of Aβ from plasma via immunoprecipitation, purification by liquid chromatography and measurement via mass spectrometry. Other groups are developing similar diagnostic testing, the Quanterix SIMOA system which, by reaction with monoclonal antibodies, determines phosphorylated Tau with a 90% sensitivity in AD [19]. A later study noted that neurofilament light chain may even be a better predictor of AD amyloid formation [20]. Even now, accuracies as high as 90% to 95% are being claimed. As these studies continue, it is probable that even further improvement can be foreseen. In this study, we have stratified the results by ApoE status, CMV IgG and CD4:CD8 ratio. These methods gave us an opportunity to assess baseline risk and then the change in risk after BCG intervention in cognitively unimpaired participants.

In the past decade, there have been several clinical trials that re-introduce non-pathogenic BCG to stimulate immune remodeling against infectious, autoimmune and allergic diseases [21,22,23]. The 100-year history of BCG with its favorable safety profile, low cost and relatively good availability makes it an attractive interventional agent for these diverse diseases.

While the primary use of BCG is for the prevention of tuberculosis [24], there is increasing evidence that BCG vaccination provides protection against non-tuberculous mycobacteria (NTM) infections; this includes resurgence of such infections after discontinuation of BCG [25,26,27]. BCG protection also extends to leprosy [28] and Buruli’s ulcer [29]. This is unsurprising as BCG, a live attenuated vaccine, shares epitopes with mycobacteria other than *M. tuberculosis* [30]. This notion, along with other evidence has led to the suggestion of a role for another zoonotic mycobacterium, *M. avium* ss. *paratuberculosis* (MAP) in AD [31]. It is notable that the ongoing BCG clinical trials for autoimmune diabetes and multiple sclerosis are both diseases associated with MAP [32].

There is increasing evidence that BCG immunization lowers overall mortality that is beyond its limited protection against TB. In part, this protection is related to increased protection by BCG against other infections including viruses such as Yellow Fever virus, human papillomavirus, respiratory syncytial virus, and SARS-CoV-2. With varying degrees of evidence, such protection has been shown in both experimental animals and humans. Such non-specific protection by BCG against non-mycobacterial infections and perhaps other non-infectious disorders include induction of: *(i)* a heterologous TH1, TH17, and CD8+ lymphocytic response and *(ii)* a non-specific memory in innate immune cells (natural killer cells, monocytes, and macrophages) through epigenetic effects and metabolic rewiring in a paradigm known as “trained immunity” [33]. Of contemporary note is the investigation of BCG in the COVID-19 pandemic; BCG benefit has been shown in both animal COVID-19 studies and humans where BCG demonstrated a “synergy” with COVID-19 vaccination [34,35,36]. 

Epidemiologic studies of intradermal BCG (as a TB vaccine) and intravesicular BCG (for bladder cancer immunotherapy) administration showing an inverse relationship with the prevalence of AD implicate only an association and not necessarily a causal effect. However, our prospective finding that intradermal BCG induces a favorable profile of a peripheral biomarker for AD is a highly promising signal. What could be the mechanism(s) by which BCG induces a favorable impact? Regardless of the inciting event for AD, it is becoming increasingly clear that damaging neuro-inflammation contributes to the neurodegeneration seen with AD [37,38]. An important player in this neuro-inflammation are microglial cells (essentially brain tissue macrophages) that become overwhelmed in their intended role in ingesting and removing the Aβ. Due to increased activation of the microglial cells in the face of their reduced capacity, there is excessive inflammation that results in neurotoxicity and neuroinflammation [3]. In addition to this ineffective pro-inflammatory state, a decrease in anti-inflammatory T regulatory cells (Tregs) contributes to the neuropathology as these cells have been shown to be protective against excessive inflammation [39]. Thus, one potential mechanism by which BCG may be protective against pathology of AD is via non-specific activation by BCG (an “off-target” effect) of both the innate and adaptive immune system that augments the ability of microglial cells in clearing Aβ. Another is that since BCG is known induce IL-2 (aka T cell growth factor) and Tregs have express high levels of the subunit of IL-2 receptor (IL-25), BCG is likely to induce Treg replication and dampen the harmful pro-inflammatory response seen with AD.

In the APP/PSI AD mouse model, BCG immunization reversed their cognitive decline but did not reduce the burden of Aβ in the brain [5]. Furthermore, the BCG vaccinated mice had increased influx of inflammatory-resolving blood monocytes into the areas of the brain with plaque pathology. While BCG also upregulated cerebral anti-inflammatory cytokines, it also down-regulated the increased splenocyte (systemic) Tregs of the AD mice back down to levels seen in the wildtype mice [5].

BCG may also afford protection against demyelinating disease such as multiple sclerosis [40] and experimental allergic encephalomyelitis (EAE) [41]. In the latter instance, intracerebral infection of BCG in a murine model of EAE resulted in decreased frequency in the brain of myelin oligodendrocyte glycoprotein-specific IL-17^+^CD4^+^ and IFNγ^+^CD4^+^ T cell responses [41].

Our finding of an AD benefit from vaccination adds to a growing list of studies that have found an association between vaccination and decreased AD risk. A relationship between Tetanus, Diptheria and Pertussis (Tdap) vaccine and AD was investigated in a retrospective review of 100,000+ medical claims from Veteran Health Administration and private sector cohorts [42]. After controlling for confounders, the researchers found a 46–53% reduction in AD among patients who had received Tdap. A similar study was undertaken to investigate an association between herpes zoster (HZ) vaccination and dementia [43]. Compared with no HZ vaccination, HZ vaccination resulted in a 31–35% risk reduction in dementia and a 25–30% risk reduction in AD. Another interesting study used a similar methodology but considered the impact of both HZ and Tdap vaccination on dementia risk [44]. This study found a significantly greater reduction in dementia risk when both vaccines were received, compared to just one or neither.

With the preceding discussion of the potential AD benefit from various vaccines, the question can rightly be posed: what about the COVID-19 vaccines. Both epidemiological and clinical studies suggest BCG vaccine has protective effects against COVID-19 [45,46]. All but two of our 49 participants received the COVID-19 vaccine. One non- COVID-19 vaccinated participant had an intermediate range APS of 37 and a follow up APS of 43. Of note, she was hospitalized for COVID-19. The other non- COVID-19 vaccinated participant had an APS of 3 with the follow up score 2. The Clincaltrials.gov site for BCG/ COVID-19 lists 29 international studies assessing BCG as a preventative against COVID-19. This includes a multicenter US study: ClinicalTrials.gov Identifier: NCT04348370. The large data sets associated with COVID-19 vaccines will undoubtedly be mined for years to come including the role that BCG may have had with COVID-19 [47].

The results of this study show the utility of the plasma amyloid-based APS particularly as it is able to identify higher risk individuals at an age when intervention, in this case BCG, can be more successfully introduced. The long history of BCG use, its strong safety profile and success in this pilot trial would make it a strong candidate for further study. Taken as a whole, the reduction of risk of cerebral amyloid as determined by an increase in plasma amyloid 42/40 ratio and decrease in APS after BCG vaccination is reported in this study. The favorable changes are statistically significant in younger participants, in those with the highest risk category, those not latently infected with CMV and those participants with a favorable lymphocyte immune risk profile (CD4:CD8 > 1.5).

## Figures and Tables

**Table 1:** Participant Demographics.

	Females	Males	*p*-Value
***n*** **(%)**	28 (57.1)	21 (42.9)	
**Age, years**			
Mean (SD)	64.3 (6.7)	65.7 (8.4)	0.52 ^a^
**Length of education, years**			
Median (IQR)	16 (14–18)	16 (16–18)	0.47 ^b^
**CMV infection, *n* (%)**			
Negative	19 (67.9%)	15 (71.4%)	0.79 ^c^
Positive	9 (32.1%)	6 (28.6%)	
**ApoE4 allele, *n* (%)**			
Negative	18 (64.3%)	9 (42.9%)	0.14 ^c^
Positive	10 (35.7%)	12 (57.1%)	
**SAGE**			
Before BCG			
Median (IQR)	22 (21–22)	22 (21.5–22)	0.70 ^b^
After BCG			
Median (IQR)	22 (22–22)	22 (21–22)	0.37 ^b^

^a^ Two-sample independent *t*-test, ^b^ Mann–Whitney test, ^c^ Chi-square test.

## Data Availability

We have applied to submit data to the alzheimer’s data initiative: www.alzheimersdata.org (accessed on 28 December 2021).
